# The Relevance of the Expected Value of the Proportion of Arabian Genes in Genetic Evaluations for Eventing Competitions

**DOI:** 10.3390/ani13121973

**Published:** 2023-06-13

**Authors:** María José Sánchez-Guerrero, María Ripollés-Lobo, Ester Bartolomé, Davinia Isabel Perdomo-González, Mercedes Valera

**Affiliations:** Departamento de Agronomía, ETSIA, Universidad de Sevilla (Spain), Carretera de Utrera Km 1, 41013 Sevilla, Spain; marriplob@alum.us.es (M.R.-L.); ebartolome@us.es (E.B.);

**Keywords:** composite breeds, equine, sport competition, variance heterogeneity

## Abstract

**Simple Summary:**

The relevance of the expected value of the proportion of Arabian genes (EV%AG) in different horse breeds participating in the eventing discipline, and the way this factor should be included in equine genetic evaluations for eventing, have not been studied in depth in the current literature. A total of 1089 horses participating in eventing competitions (8862 participation records) were used for this study. The significance of the EV%AG in the different scores obtained was studied, and was found to be significant for the three exercises that make up the discipline of eventing (dressage, show jumping and cross-country). Five genetic models were computed to study the importance of the EV%AG in the pedigree of 10,375 horses. The best-fitted models following the DIC criterion were those including age as covariate, sex, breed, level, EV%AG and event as systematic effects, rider, animal and residual as random effects and variance heterogeneity, concluding that the best way to model the EV%AG effect seems to be by considering the variance heterogeneity. Dressage and show jumping heritability ranged from 0.10 in approach C to 0.21 in D. The estimated heritability for the cross-country trait oscillated less, between 0.07 and 0.01 in approach D.

**Abstract:**

The Arabian horse is a generally reliable sport horse, and continues to be a remarkable endurance horse, so the relevance of the expected value of the proportion of Arabian genes (EV%AG) in horses participating in eventing could be a relevant factor. A total of 1089 horses participating in eventing (8866 records) were used. A GLM revealed that the EV%AG was significant in dressage, show jumping and cross-country. A BLUP genetic evaluation was computed with five genetic models (without the EV%AG (0) using as a covariate (A), as a fixed effect (B), with variance heterogeneity, and in genetic groups without (C) and with (D)). Dressage heritability ranged from 0.103 to 0.210, show jumping ranged from 0.117 to 0.203 and cross-country ranged from 0.070 to 0.099. The lowest DIC value was used as a criterion of fitness. The best fits (those which included variance heterogeneity) showed fewer than two points of difference in DIC values. The highest average estimated breeding value in dressage, show jumping and cross-country was found for horses with an expected value of the proportion of Arabian genes of 0%, ≥1% to <25%, and 100%, respectively. Therefore, the best way to model the EV%AG effect seems to be by considering the variance heterogeneity.

## 1. Introduction

Horses have been used for centuries for warfare, transportation or farming, in all of which physical performance is particularly relevant. In the 20th century, certain horse breeds have increasingly been used and bred for competitive sports disciplines such as show jumping, dressage and eventing [1].Composite breeds in any animal species are created to take advantage of the mixture of abilities among progeny [2,3], and as the genetic contributions coming from the parents may vary, composite horse breeds are created by crossing two or more breeds to obtain the desired combination of traits and characteristics [4,5,6,7]. Generally, these composite horse breeds perform better in certain equine competitions such as eventing [8]. Eventing is the most complete combined competition of the three Olympic equestrian disciplines, requiring the participant to have considerable experience and an expert knowledge of the physical and psychological characteristics of the horse [9]. Moreover, eventing requires the horse to display a wide range of complex aptitudes related to its intelligence and progressive training. The discipline consists of three different exercises: dressage, show jumping and cross-country, all performed by the same rider and horse [10].

The Arabian horses are commonly believed to be among the oldest and most influential horse breeds in the world [11]. Historical records show that the Bedouins (the original breeders of the horse in the Arabian Desert) used traditional methods to maintain the purity of the Arabian horse. These included avoiding any mating between Arabian horses and non-Arabian horses and maintaining strictly separated strains [12]. The Arabian horse remains a remarkable sports horse to this day, showing particular prowess as a racehorse and an endurance horse [13]. It is distinguished by its flexibility, maneuverability, resistance and lightness [14]. Today, this breed stands out as the best of all breeds in the equestrian discipline of endurance, although in other disciplines it shows certain limitations.

Selection processes and mating are influenced by conscious decisions, usually with the sole intention of achieving short-term aims in intensively managed domesticated species [15]. In most of the horse breeds dedicated to sport competitions, the main objective of selection is to obtain good results at the highest levels of competition, in whatever discipline they take part in [16]. In Spain, a number of horse breeds participate in eventing competitions, in particular the Caballo de Deporte Español, Anglo-Arabian horses, European Sport horses (Belgian Sport horse, Belgian Warmblood horses, Hanoverian horses, Holsteiner horses, Irish Sport horses, Koninklijk Warmbloed Paard Nederland horses, Westphalian horse and Zangersheide horses), Arabian horses, Selle Français, Pura Raza Española horses, Thoroughbreds and Hispano-Arabe horses. Most of the breeds used are composite breeds which could have recent Arabian horse ancestors in their pedigree (except the Pura Raza Española horse and the Thoroughbred). Previous studies have reported that Arabian horses were bred mainly for the endurance discipline [17], which shares with eventing the aptitudes of resilience and agility, both of which are required for horses to compete successfully [18]. Thus, the presence of Arabian genes in any horse breed can have a significant impact on performance, as these physical and physiological characteristics can give the horse an advantage over other horse breeds, which is therefore highly beneficial for breeders.

In spite of this, while many authors have studied the impact of genetics on performance in different equine sport competitions [19,20,21,22,23,24], little research has been carried out into the relevance of the expected value of the proportion of Arabian genes (EV%AG) in different horse breeds participating in the discipline of eventing, or the way in which this factor can be included in equine genetic evaluation. Understanding how the EV%AG influences a horse’s potential success in competitive events could help breeders to make decisions in their mating plans and decide where to invest their efforts and resources to maximize performance results. Therefore, the specific aim of this work was to model the EV%AG effect and to estimate the genetic parameters and breeding values, using five different genetic models to test its suitability for genetic selection.

## 2. Materials and Methods

### 2.1. Description of Eventing: Dressage, Show Jumping and Cross-Country Exercises

Eventing competitions consist of three different exercises, with the winning horse having the best average total score over the three.

In the dressage exercise, the horse has to make a pre-arranged series of dressage movements on a marked track in which different variables are evaluated by two or three judges with a score ranging from 1 to 10. The scores for the different variables are then averaged and calculated on a scale of 1–150. 

The show jumping exercise consists of an obstacle contest on a track, with a fixed time limit. The score is calculated from the penalties incurred by each participant for faults in jumping over the obstacles, added to any penalties they may have obtained for exceeding the time allowed: the fewer penalty scores obtained, the better the performance. All the scores are converted to positive scores by assigning the highest score (125 points) to the animals with the best performance (0 penalties). 

The cross-country exercise is an obstacle race on a country track. The score for this exercise is also based on penalty scores which are then converted by assigning a score out of 200 to the performance, according to the number of penalties.

### 2.2. Description of the Database and Expected Value of the Proportion of Arabian Genes

A total of 409 Spanish eventing competitions celebrated between 2004 and 2021 were used for the analysis. These competitions had 8 different levels of difficulty, with each competition involving one or more of these levels of difficulty.

For the purposes of the present study, the dataset was reduced by deleting animals with both parents unknown or with any missing records in any exercise. After this step, the final database included 8862 records of 1089 horses (443 mares, 560 stallions and 86 geldings) with an average age of 7.48 ± 3.30 years old. The final pedigree dataset contained 10,375 horses (6208 mares, 4081 stallions and 86 geldings), with horses belonging to eight different breed groups (Table 1). The EV%AG of an individual horse is calculated based on its pedigree, which involves going back to the last known generation (a minimum of 3 and a maximum of 16 generations). To calculate it, we assumed that all Arabian horses had 100% Arabian EV%AG, while Anglo-Arabian and Hispano-Arabe horses had a proportion of Arabian genes corresponding to the information included in their studbook. Once the pedigree is reconstructed, this information is calculated by considering that the foals of two horses would have the sum of the expected value of the proportion of Arabian genes of both parents divided by two. Logically, in a generic way, it also follows that a foal (AB) has the average of the genes of the parents (A and B). By this reckoning, Arabian horses had an EV%AG of 100, Pura Raza Español and Thoroughbred had an EV%AG very close to 0, and other composite breeds had an EV%AG which ranged between 0 and 99%.

### 2.3. Statistical Analyses

First, a basic descriptive statistical analysis of the three eventing exercises score was carried out, and the significance of the EV%AG factor (5 levels: 0% (440 horses); >0% to <25% (276 horses); ≥25% to <50% (243 horses); ≥50% to <100% (59 horses) and 100% Arabian horses (71 horses)) was studied, together with its influence on the scores obtained on each exercise (dressage, show jumping and cross-country), using a multivariate general linear model (GLM). All the other systematic factors were also studied in a multivariate GLM. Next, a Fisher post hoc test was carried out to highlight the differences between the least-squares means score for the three eventing exercises, depending on the EV%AG effect. Phenotypic Pearson correlations were also made among the three eventing exercises studied. Statistical analyses were performed using Statistics for Windows software v.11 [25].

### 2.4. Genetic Model

The estimation of the genetic parameters was carried out with a BLUP genetic evaluation based on a multivariate animal model, with the scores obtained for dressage, show jumping and cross-country as traits. Five different genetic models were compared using each trait, and genetic parameters were estimated for all of them. The different approaches (Table 2) could include as linear covariates the age or the EV%AG, and as fixed effects, sex (male, female and geldings), breed (8 levels, Table 1), level of the competition (8 levels), event (409 levels) and the EV%AG with 5 levels: (0%; >0% to <25%; ≥25% to <50%; ≥50% to <100% and 100% Arabian horses). Additive genetic and residual effects were included as random factors, besides the rider effect, with 583 levels.

A heterogeneous variance model was used to allow the residual variance to be estimated, based on EV%AG. Such differences in variance may occur if the mating of the horses is not planned in a natural way. Here, residual variances can be considered heterogeneous, and can be divided according to the EV%AG into five subclasses for all the horses studied. This involves the classification of arbitrary subclasses, within which the variance is assumed to be constant, and where the change in the residual variance is continuous over time [26]. These differences can also be found in genetic groups. Not taking into account the fact that that base populations are genetically heterogeneous, and thus split into different ‘genetic groups’, may lead to biased parameter estimates, especially for additive genetic variance. To avoid such biases, we have proposed animal models divided into groupings containing more than one genetic group [27]. Therefore, to shed light on the best way to genetically select animals for eventing with different EV%AG values, models A, B and D fitted genetic groupings from different origins, included 8 genetic groups according to combinations of the EV%AG (0%, 0–50%, 51–99% and 100%) with the year of birth (born before or since 1960). The use of genetic groups is indicated when there are different populations of origin, with, presumably, different means [28].

All of the models were analyzed using a Bayesian approach via Gibbs sampling with the GIBBSF90+ module of the BLUPF90 software [29]. The Gibbs sampler was run for 100,000 rounds, with the first 20,000 considered as burn-in and then every 100th sample saved for later analysis. Posterior means and standard deviations were calculated with POSTGIBBSF90 software [29] to obtain estimates of variance components and estimated breeding values (EBV). Convergence of the posterior parameters was assessed by visual inspection of trace plots of posterior distributions generated by the Coda R package [30]. The equation in matrix notation for the model (assuming homogeneity of the residual variance) to be solved for a hypothetical trait considering all of the possible random effects was:

*y_i_* = x*_i_*b + z*_i_*u + w*_i_*r + *e_i_* (1)

The equation in matrix notation for the model (assuming heterogeneity of the residual variance) to be solved for a hypothetical trait considering all of the possible random effects was:

*y_i_* = x*_i_*b + z*_i_*u + w*_i_*r + *e*^1/2(x*_i_*b∗+z*_i_*u∗+w*_i_*r∗)^*_ε__i_* (2)

where *y* is the vector of observations, * indicates the parameters associated with residual variance, b and b^∗^ are vectors of the systematic effects, u and u^∗^ are vectors of the additive genetic effects, r and r^∗^ are vectors of a rider random effect, and x*_i_*, z*_i_*, and w*_i_* are incidence vectors for systematic, animal, and rider random effects, respectively. Finally, *εi*∼*N*(0,1). The genetic effects u and u^∗^ were assumed to be Gaussian.

The lowest deviance information criterion (DIC) value [31] was used as a criterion of fitness. Estimated breeding values were typified (on a scale of 80–120) and reliability was calculated as 1 − (PEV/σ_u_) for the heterogeneity linear model with genetic groups. In order to analyze the distribution of the animals with best estimated breeding values using genetic model D, only the 20% lower and upper breeding values with a reliability ≥ 50 were used here.

## 3. Results

The mean score of the three traits studied ranged from 99.99 to 187.47 for both dressage and cross-country (Table 3). The show jumping exercise had a coefficient of variation of 6.03%, and a lower range of scores.

The generalized linear model analysis showed that the three equestrian exercises that make up the eventing competition showed statistically significant (*p* < 0.05) differences according to the horse’s EV%AG (Table 4), sex (only for dressage), breed, level, event and age. Furthermore, Arabian horses showed the best scores for dressage, with between 1 and 99% of EV%AG for the cross-country discipline. Finally, for show jumping, the highest scores were obtained by horses with an EV%AG ranging between 25 and 50%.

**Figure 1:**
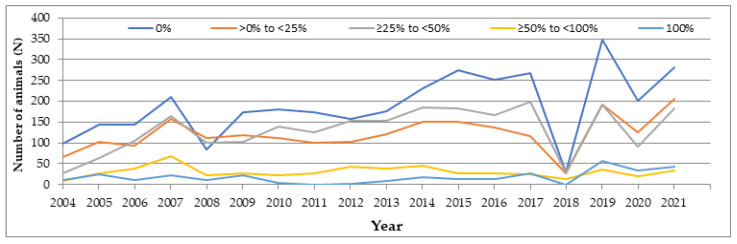
Number of horses participating in eventing competitions according to the expected value of the proportion of Arabian genes by year.

In spite of Table 4 indicating that horses with any degree of EV%AG showed better results in the three equestrian exercises that make up the eventing competition than horses with 0% of EV%AG, the evolution of horses participating in these three exercises from 2004 to 2021, according to the EV%AG, indicated that horses with no EV%AG had a higher number of participations (Figure 1). Therefore, the breed of the horses which participated more could include horses of all breed groups except the Anglo-Arabian horse, Arabian horse and Hispanic-Arabian horse, due to the fact that they would always have some Arabian genes. In 2008 and 2018, horses that had an EV%AG between 1% and 50% were those which participated more in eventing competitions in Spain. Arabian horses were the least used until 2019, while after that, the least-used horses were those with 50% to 99% EV%AG.

The best-fitted models were C and D, with fewer than 2 DIC points of difference (Table 5). The heritabilities were medium-low and close between the three traits studied (dressage, show jumping and cross-country) in the different models. Heritability for the dressage exercise ranged from 0.10 in approach C (100% EV%AG) to 0.21 in approach D (≥50% to <100% EV%AG), while for the show jumping exercise, it ranged from 0.12 in approach C (100% EV%AG) to 0.20 in the approaches without heterogeneity (0, A and B). The estimated heritability for the cross-country exercise oscillated between 0.07 (approach C and D, 0% and >0% to <25% EV%AG) and 0.10 in the D approach without heterogeneity (≥25% to <50% EV%AG). 

In our study, the genetic correlations among the three equestrian exercises that make up the eventing competition (Table 6), according to the five different genetic models used, were positive, showing moderate values (from 0.23 for dressage and cross-country with approach C to 0.50 for show jumping with cross-country genetic models 0 and C). 

Residual correlations (Table S1) were low, oscillating from 0.113 for dressage and cross-country with approach C to −0.168 for show jumping with the dressage genetic model D. The genetic correlations were similar within the five genetic models in all the combinations of exercises, while the phenotypic correlations were substantially lower than the genetic correlations.

The highest average estimated breeding values (Table 7) were found in horses with an EV%AG near to 0% (dressage), >0% <25 (show jumping) and 100% (cross-country).

## 4. Discussion

Eventing performance comparisons among horses with different EV%AG are justified, due to the fact that the genetic differences among breeds or strains are large in relation to the genetic variation within the same breed. Horses with an EV%AG ranging from 1 to 99% showed a better performance in Spanish eventing competitions, which could be related to the hybrid vigor effect resulting from crossbreeding. On the other hand, horse breeds have been selected to produce animals that best suit the equestrian discipline they participate in, and this holds true for pure breeds as well as for composite breeds [32]. In fact, in composite breeds of domestic horses, a large part of the widely described hybrid vigor is linked to physical performance, which is targeted to fit human needs and uses [33], and can be seen in improved physical and physiological characteristics, such as strength or endurance [34]. These differences constitute an important potential source of genetic improvement for breeds in performance [35]. Cases of increased fitness and a selective advantage over the parents in composite breeds have been documented in many taxa [36]. This phenomenon, generally considered as the result of heterozygosis in hybrids [37], is known as hybrid vigor, or heterosis, and is usually measured by the capacity of hybrids to expand their ecological range and outperform their parent species under natural conditions [38]. Furthermore, composite breeding involving different breeds is frequently used to produce riding horses, and many outstanding show jumpers and eventing horses have resulted from successful crosses [39]. Previous studies have also reported how Arabian and Anglo-Arab horses performed better in eventing competitions, compared to other horse breeds such as the Pura Raza Español [8,20]. Despite this, most of the horses used in eventing competitions in Spain from 2004 to 2021 have been those with 0% EV%AG.

According to the least-squares means (Table 4), horses with 1% to 99% EV%AG perform better in dressage, eventing and show jumping, but with differences relating to the percentage of genes. Such horses (mainly sport composite breeds and Anglo-Arab horses) are used worldwide for Olympic equestrian disciplines (dressage, show jumping and eventing) [16]. Most of the EV%AG horses are bred to perform the three types of exercises [16]. Surprisingly, horses with 0% EV%AG (such as Pura Raza Español horses) that participate in eventing do not excel in their traditional subtests such as exercise (dressage).

In a genetic evaluation, fitting different additional random effects besides the residual and additive genetic effects has always been a topic of debate [22,40]. Models C and D contained heterogeneous variance and were the best fit, which confirms that heterogeneous models tend to give preferable results to those of the classic homogeneity models [41,42,43]. It could be due to these models collecting the variance from the EV%AG in the three exercises studied. The highest variability of the phenotype score for 100% EV%AG (dressage and show jumping) and 0% EV%AG (cross-country) within the different percentages of blood, coincides with the highest residual variance in the variance heterogeneity models. In contrast, in our study, including or not including the genetic group was not a relevant factor. Heritability value ranges were similar to those previously estimated in sport horses (0.03 cross-country to 0.26 show-jumping) [8,44,45]. Dressage exercise heritability estimates are comparable with estimates both for the individual competition discipline in Spain (Pura Raza Español) and for international estimates (Dutch Warmblood and sport horse), namely 0.10–0.21 [40,44,46,47]. Similarly, show jumping heritability estimates are comparable to international estimates for competition data (Dutch Warmblood and sport horses), ranging from 0.12 to 0.26 [21,44,47,48]. Finally, cross-country heritability estimates were lower, ranging from 0.03 to 0.09 (crossbreed and sport horses), which was also in concordance with previous studies [8,45].

In general, the disadvantage of considering a multiple-trait breeding objective such as eventing competitions is that the genetic improvement per trait (in absolute biological units) can be considerably lower when compared with single-trait breeding goals, so the genetic correlations can indicate the extent to which the traits of interest can be improved simultaneously [8]. Our results were similar to previous studies such as Ricard and Chanu [46], who reported moderate genetic correlations between eventing and other equestrian disciplines such as show jumping (0.45) or dressage (0.58). However, our study differs from the previous studies which obtained a genetic correlation between show jumping and cross-country exercise close to one, with dressage showing a better correlation with cross-country than with show-jumping [17,48,49,50].

In composite breeds with Arabian genes, the selection of mare and stallion for breeding is influenced by the stud preferences (often including, in the case of breeding horses for eventing competition, the expected value of the proportion of Arabian genes). Consequently, this choice is made to improve the global performance of the future foal having a relevant effect on other genetic parameters. In dressage, the average breeding values were higher in horses with no expected value of the proportion of Arabian genes than in the other horses, which could be due to the fact that most of these animals belonged to the Pura Raza Española breed, which is selected to perform best in dressage competitions [51,52]. These favorable Pura Raza Española genetic values contrast with the performance results obtained in eventing dressage exercises. One hypothesis to consider would be that the Pura Raza Española horses used to perform in eventing were selected for their aptitude in the three exercises (not focusing only on dressage), while the Pura Raza Española horses used as breeders of other composite sport horses were mainly selected to provide genetic quality in dressage exercises.

In contrast, the highest estimated breeding values for the cross-country exercise were found in Arabian horses. As reported previously, these animals are usually bred for the endurance discipline [17], which consists of running long distances over tracks in open fields. These competition conditions could be considered similar to those found in the cross-country exercise of the eventing competition. Thus, the adaptation of this breed to this type of performance could provide these animals with a physical and physiological advantage over other horse breeds. These positive genetic values contrast with the performance results obtained in eventing cross-country exercises. One hypothesis to consider would be a similar one to the case of the Pura Raza Español breed, in other words that Arabian horses used as breeders of other composite sports horses were mainly selected to provide genetic quality in the cross-country exercise.

## 5. Conclusions

According to our results, the best way to model the expected value of the proportion of Arabian gene effect to estimate genetic parameters and to determine the animals’ breeding value for the eventing discipline seems to be by taking into account the heterogeneity of variance.

## Figures and Tables

**Table 1:** Number of horses participating in eventing by breed, with the average expected value of the proportion of Arabian genes.

Breed	N	Average Expected Value of the Proportion of Arabian Genes (%)
Caballo de Deporte Español (CDE)	439	4.60
Anglo-Arabian Horse (AA)	330	33.36
European Sport Horses (ESH)	124	2.05
Arabian Horse (AH)	71	100
Selle-Français (SF)	51	0.28
Pura Raza Española (PRE)	32	0
Thoroughbred (TH)	25	0
Hispano-Arabe Horse (HA)	17	43.01

**Table 2:** The five genetic models used to estimate genetic parameters modeling the expected value of the proportion of Arabian genes.

Model	Covariate	Systematic Effect	Random Effect	Heterogeneity	Genetic Group
0	Age	Sex Breed Level Event	Additive Rider Residual	No	No
A	Age EV%AG	Sex Breed Level Event	Additive Rider Residual	No	Yes
B	Age	Sex Breed Level Event EV%AG	Additive Rider Residual	No	Yes
C	Age	Sex Breed Level Event EV%AG	Additive Rider Residual	EV%AG group	No
D	Age	Sex Breed Level Event EV%AG	Additive Rider Residual	EV%AG group	Yes

EV%AG: Expected value of the proportion of Arabian genes.

**Table 3:** Basic statistical description of the three eventing exercises: dressage, show jumping and cross-country score.

Eventing Exercise	N	Mean ± s.e.	Range	C.V.
Dressage	8862	99.99 ± 0.12	51–150	11.39%
Show-jumping	8862	119.71 ± 0.08	40–125	6.03%
Cross-country	8862	187.47 ± 0.20	21–200	10.18%

s.e.: standard error; C.V.: Coefficient of variation.

**Table 4:** Generalized Linear Model (GLM) and post hoc analysis (Fisher LSD) of the three equestrian exercises that make up the eventing competition, with the expected value of the proportion of Arabian genes.

Equestrian Discipline	Expected Value of the Proportion of Arabian Genes					*p*-Value
	0	>0 to <25	≥25 to <50	≥50 to <100	100	
	Least-Squares Means					
Dressage	99.60 ^a^	99.99 ^ab^	100.29 ^ab^	100.56 ^a–c^	101.37 ^c^	0.019
Show jumping	119.40 ^a^	119.93 ^b^	120.03 ^b^	119.82 ^ab^	118.98 ^a^	0.036
Cross-country	185.08 ^a^	186.78 ^b^	190.82 ^c^	191.11 ^c^	186.67 ^ab^	0.000

Different superscript letters (a, b and c) indicate a statistically significant difference between groups (*p* < 0.05).

**Table 5:** Heritability of the three equestrian exercises that make up the eventing competition (dressage, show-jumping and cross-country scores) in the five complementary approaches (0, A, B, C and D), together with the DIC value.

Models			σ_u_			σ_r_			σ_e_			h^2^	DIC
			Mean	Median	HPD 95%	Mean	Median	HPD 95%	Mean	Median	HPD 95%		
Dressage	0		12.07	12.030	10.02–14.48	14.58	14.55	11.24–18.07	35.83	35.82	34.67–37.13	0.193	158,850.17
	A		12.19	12.100	10.02–14.43	13.79	13.70	10.77–17.35	35.78	35.78	34.70–37.05	0.197	158,823.34
	B		12.12	12.060	10.02–14.30	13.86	13.82	10.51–17.10	35.77	35.77	34.61–36.97	0.196	158,824.44
	C	0%	11.12	11.08	9.18–12.84	13.11	12.99	10.04–16.23	32.48	32.49	30.73–34.26	0.196	158,611.69
		>0%–<25%							32.46	32.43	30.31–34.69	0.196	
		≥25%–<50%							39.83	39.85	37.28–42.08	0.174	
		≥50%–<100%							29.91	29.75	25.84–34.11	0.205	
		100%							83.95	83.17	68.47–98.68	0.103	
	D	0%	11.61	11.62	9.51–13.73	13.06	12.95	9.99–16.21	32.55	32.55	30.72–34.23	0.203	158,611.27
		>0%–<25%							32.65	32.60	30.52–34.86	0.203	
		≥25%–<50%							40.31	40.15	37.58–42.43	0.179	
		≥50%–<100%							30.45	30.30	26.35–34.75	0.210	
		100%							84.72	84.09	70.39–100.70	0.106	
Show-jumping	0		8.89	8.83	7.31–10.60	1.87	1.81	0.97–2.77	33.70	33.65	32.64–34.81	0.200	158,850.17
	A		9.08	9.05	7.40–10.85	1.88	1.82	0.92–2.82	33.66	33.65	32.57–34.73	0.203	158,823.34
	B		9.10	9.04	7.48–10.84	1.88	1.84	0.92–2.73	35.77	35.77	34.61–36.97	0.204	158,824.44
	C	0%	7.84	7.82	6.33–9.24	2.01	1.97	1.17–2.94	34.27	34.25	32.40–36.07	0.178	158,611.69
		>0%–<25%							30.73	30.71	28.78–32.86	0.193	
		≥25%–<50%							30.69	30.69	28.69–32.51	0.193	
		≥50%–<100%							44.72	44.45	38.81–50.35	0.144	
		100%							56.92	56.77	47.06–66.70	0.117	
	D	0%	8.13	8.06	6.57–9.69	1.90	1.88	1.06–2.73	34.369	34.37	32.54–36.24	0.183	158,611.27
		>0%–<25%							30.98	30.99	28.69–32.83	0.198	
		≥25%–<50%							30.67	30.66	28.62–32.38	0.200	
		≥50%–<100%							45.787	45.62	39.70–51.63	0.146	
		100%							56.15	55.86	46.54–65.43	0.123	
Cross-country	0		32.57	32.51	22.76–40.53	44.23	43.65	32.31–60.27	35.829	35.82	34.67–37.13	0.090	158,850.17
	A		33.47	33.09	24.27–42.27	44.61	44.11	32.33–57.93	281.20	281.10	272.90–290.70	0.093	158,823.34
	B		33.44	33.39	25.02–43.31	43.77	43.23	30.83–58.62	281.12	281.00	272.50–289.80	0.093	158,824.44
	C	0%	27.68	27.54	19.94–36.47	40.64	40.00	28.60–55.98	306.09	305.90	291.30–324.20	0.074	158,611.69
		>0%–<25%							324.72	324.70	305.50–345.80	0.070	
		≥25%–<50%							225.05	224.90	212.30–238.80	0.094	
		≥50%–<100%							259.52	258.40	229.90–293.90	0.084	
		100%							267.32	265.60	220.20–313.30	0.082	
	D	0%	29.14	28.92	19.97–37.35	40.85	40.20	27.28–54.36	306.44	306.30	289.40–322.70	0.077	158,611.27
		>0%–<25%							324.44	324.10	305.10–346.20	0.074	
		≥25%–<50%							225.42	225.40	213.20–239.40	0.099	
		≥50%–<100%							260.83	259.60	229.90–293.50	0.088	
		100%							269.25	268.40	220.10–314.50	0.086	

HPD 95% = interval 95% Highest Probability Density; σ_u_ = additive variance; σ_r_ = rider variance; σ_e_ = residual variance; DIC = deviance information criterion and h^2^ = heritability.

**Table 6:** Genetic and phenotypic correlations of the three equestrian exercises that make up the eventing competition (dressage, show jumping and cross-country scores) in the five complementary approaches (0, A, B, C and D).

Model			Show Jumping	HPD 95%	Cross-Country	HPD 95%
Genetic correlation	0	Dressage	0.268	0.136–0.381	0.241	0.075–0.386
	A		0.264	0.146–0.386	0.269	0.129–0.431
	B		0.259	0.135–0.388	0.271	0.125–0.430
	C		0.293	0.193–0.392	0.232	0.113–0.355
	D		0.260	0.157–0.358	0.247	0.120–0.380
Phenotypic correlation			0.107		0.083	
Genetic correlation	0	Show jumping			0.497	0.356–0.643
	A				0.479	0.335–0.610
	B				0.474	0.342–0.620
	C				0.477	0.351–0.603
	D				0.500	0.372–0.634
Phenotypic correlation					0.137	

HPD 95% = interval 95% Highest Probability Density.

**Table 7:** Number of animals (and percentage of participants in each group), and average Estimated Breeding Value (EBV) of horses placed in the top 20% percentile, with a reliability ≥ 50%.

EV%AG	Dressage		Show Jumping		Cross-Country	
	N° Horses (%)	EBV	N° Horses (%)	EBV	N° Horses (%)	EBV
0%	17 (3.86%)	105.44	22 (5%)	107.69	27 (6.14%)	107.04
>0%–<25%	16 (5.80%)	104.67	17 (6.16%)	107.97	19 (6.88%)	108.84
≥25%–<50%	12 (4.94%)	104.60	12 (4.94%)	107.85	10 (4.12%)	106.61
≥50%–<100%	7 (11.86%)	103.70	5 (8.47%)	107.81	2 (3.39%)	106.07
100%	8 (11.27%)	104.05	4 (5.63%)	106.78	2 (2.82%)	111.49

% of horses in the total of each of the groupings based on expected value of the proportion of Arabian genes (EV%AG); N° = number and EBV = estimating breeding value.

## Data Availability

www.rfhe.es (accessed on 3 December 2022).

## Supplementary Materials

The following supporting information can be downloaded at https://www.mdpi.com/article/10.3390/ani13121973/s1, Table S1: Residual correlations of the three equestrian exercises that make up the eventing competition (dressage, show-jumping and cross-country scores) in the five complementary approaches (0, A, B, C and D).
