# Supplementary material for: The Relevance of the Expected Value of the Proportion of Arabian Genes in Genetic Evaluations for Eventing Competitions
Source: Animals (Basel). 2023 Jun 13;13(12):1973. doi: 10.3390/ani13121973 (PMC10295017; doi:10.3390/ani13121973)
Supplement: Supplementary file 1 [file animals-13-01973-s001.zip › animals-2352786-supplementary.pdf]

animals MDPI

Table S1. Residual correlations of the three equestrian exercises that make up the eventing competition (dressage, show-jumping and cross-country scores) in the five complementary approaches (0, A, B, C and D).

	Models		Show-jumping	Cross-country	
Residual correlation		0	0.0186	0.0500	Dressage
		A	0.0190	0.0494	
		B	0.0193	0.0487	
	C	0%	0.0336	0.0430	
		>0%-<25%	-0.0118	0.0848	
		≥25%-<50%	0.0195	0.0146	
		≥50%- <100%	0.0520	0.1130	
		100%	-0.0344	0.0426	
	D	0%	0.0346	0.0422	
		>0%-<25%	-0.0123	0.826	
		≥25%- <50%	0.0226	0.0166	
		≥50%- <100%	0.0603	0.116	
		100%	-0.168	0.0376	
		0		0.0712	Show-jumping
		A		0.0715	
		B		0.0711	
	C	0%		0.0861	
		>0%- <25%		0.0725	
		≥25%- <50%		0.0537	
		≥50%- <100%		0.0520	
		100%		0.0405	
	D	0%		0.0881	
		>0%- <25%		0.0700	
		≥25%-<50%		0.0554	
		≥50%-<100%		0.0512	
		100%		0.0404	
